# Intraprocedural Thromboembolism From Infective Endocarditis With Immediate Endovascular Mechanical Thrombectomy: A Case of Remarkable Timing

**DOI:** 10.7759/cureus.78694

**Published:** 2025-02-07

**Authors:** Diwas Gautam, Daryl E Morrison, Michael T Bounajem, Lyska L Emerson, Ramesh Grandhi

**Affiliations:** 1 Neurosurgery, University of Utah Health, Salt Lake City, USA; 2 Pathology, University of Utah Health, Salt Lake City, USA

**Keywords:** cerebral angiogram, infectious intracranial aneurysm, infective endocarditis, intraprocedural large-vessel occlusion, thrombectomy

## Abstract

Infectious intracranial aneurysms and embolic strokes are common neurological complications in patients with infective endocarditis. Diagnostic cerebral angiograms are often performed prior to cardiac valve repair if an infectious intracranial aneurysm or intracranial hemorrhage is suspected.

We report a unique case of a patient with endocarditis who experienced an embolic stroke that resulted in large-vessel occlusion (LVO) of the middle cerebral artery during a diagnostic cerebral angiogram. Upon detection of the intraprocedural LVO, endovascular mechanical thrombectomy (EVT) was immediately performed using a stent retriever and the contact aspiration technique.

To our knowledge, this case is unique in both its pathology and remarkable timing. This case provides further evidence supporting the efficacy of EVT for treating LVO secondary to infective endocarditis.

## Introduction

Infective endocarditis (IE) can lead to neurovascular complications, such as infectious intracranial aneurysms (IIAs) and embolic strokes [1-3]. IIAs significantly increase the risk of intracerebral hemorrhage, which can result in severe morbidity or mortality [1,4]. Anticoagulation therapy may further elevate the risk of intracerebral hemorrhage in the presence of IIAs, and cerebral angiography is commonly performed before cardiac surgery to assess for infectious aneurysms [4,5]. In addition, embolic stroke accounts for up to 50% of IE-related complications [3,6], with approximately 40% involving the middle cerebral artery (MCA) [6,7]. Thrombolytic therapy increases the risk of hemorrhage, complicating the treatment of acute ischemic stroke secondary to IE, and there is no consensus regarding the safety and efficacy of endovascular thrombectomy for this condition [3]. We present a case in which a patient developed a blood clot during a diagnostic cerebral angiogram for IIA screening, leading to MCA occlusion. The large-vessel occlusion (LVO) was promptly identified, and mechanical thrombectomy successfully revascularized the vessel.

## Case presentation

**Figure 1:**
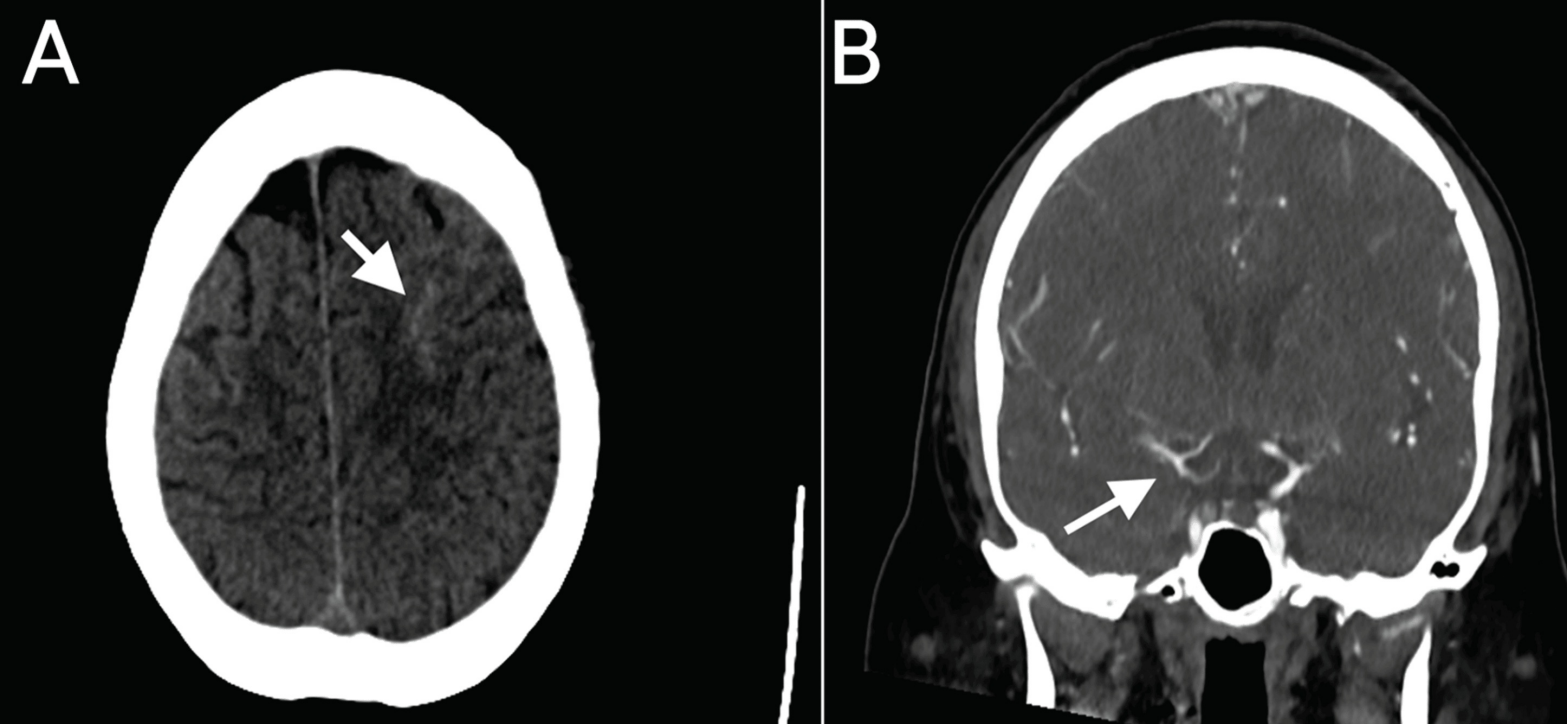
: (A) Noncontrast axial CT of the head demonstrating left frontal subarachnoid hemorrhage (arrow). (B) Coronal CT angiogram demonstrating mild irregularity in the right middle cerebral artery/anterior cerebral artery (arrow) but no evidence of vessel occlusion. CT: computed tomography.

A 51-year-old woman with a past medical history of bioprosthetic aortic valve replacement and coronary artery bypass graft surgery was found unconscious at her house and urgently transported to a nearby hospital. The patient’s vital signs indicated shock, and computed tomography imaging of her head revealed a subarachnoid hemorrhage (Figure 1A). She was subsequently transferred to our institution for higher-level care. Upon admission to the cardiovascular service, an echocardiogram showed 2-cm vegetation on her bioprosthetic aortic valve. The patient presented in a somnolent state, intermittently moaning and responding to commands by opening her eyes. Examination of cranial nerves II-XII was unremarkable. A computed tomography angiogram of the patient’s head revealed mild irregularity of the MCA/anterior cerebral artery and a slightly increased hyperdensity in the left frontoparietal region, consistent with acute subarachnoid hemorrhage (Figure 1B). Given the subarachnoid hemorrhage in the setting of IE, there was a concern for IIA, and a diagnostic cerebral angiogram was requested before proceeding with heart valve surgery.

**Figure 2:**
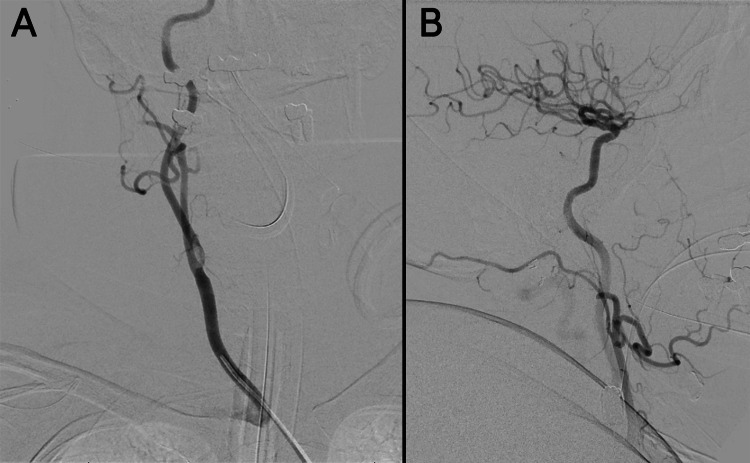
Cerebral angiograms showing the (A) anteroposterior view of the craniocervical run from the right common carotid artery showing no evidence of extracranial internal carotid artery stenosis and the (B) lateral view of the initial cerebral angiogram revealing patency of the right middle cerebral artery branches.

**Figure 3:**
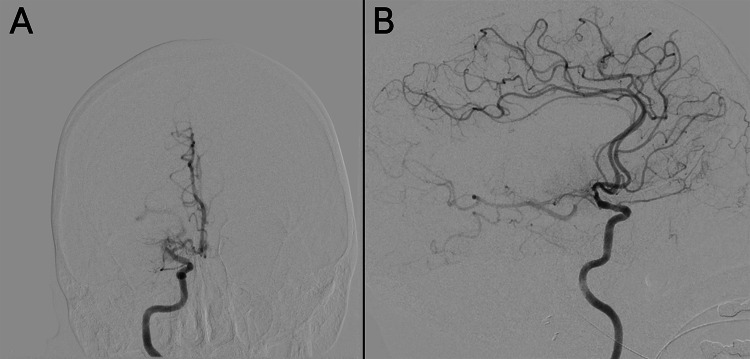
Anteroposterior (A) and lateral (B) views of the cerebral angiography revealing right M1 occlusion.

**Figure 4:**
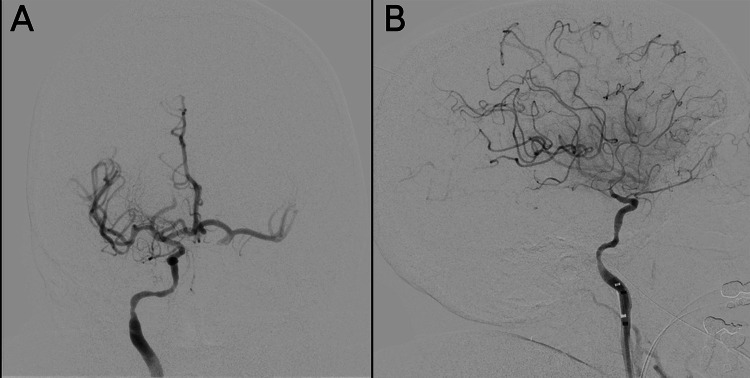
Anteroposterior (A) and lateral (B) views of the cerebral angiography revealing filling of the right M1 branches after thrombectomy Thrombolysis in Cerebral Infarction grade 3 reperfusion. This indicated complete revascularization of the affected vascular territory.

The patient was transferred to the neuroangiography suite, and a 5F Bern Select diagnostic catheter (Boston Scientific, Marlborough, MA, USA) was advanced through the aorta and brachiocephalic trunk to catheterize the right common carotid artery. A craniocervical run from the right common carotid artery showed no evidence of extracranial internal carotid artery (ICA) stenosis and confirmed the patency of the right MCA branches (Figure 2). The right ICA was selected under roadmap guidance to allow for a dedicated intracranial angiogram to evaluate the vessels for IIAs. The intracranial run revealed a proximal right MCA M1 segment occlusion (Figure 3). After identifying the LVO, preparations were made for endovascular mechanical thrombectomy (EVT). The 5F femoral sheath was exchanged for an 8F sheath. A 0.087-inch Walrus balloon guide catheter (Q’Apel Medical, Fremont, CA, USA) was advanced over a 5F Berenstein Neuron Select catheter (Penumbra Inc., Alameda, CA, USA) and a 0.035” Glidewire (Terumo Inc., Tokyo, Japan) to catheterize the right ICA. A system consisting of an Esperance 6F 132-cm aspiration thrombectomy catheter (Wallaby Medical, Laguna Hills, CA, USA) and 6.5 × 45 mm EMBOTRAP III (Cerenovus, Fremont, CA, USA) was used to achieve a Thrombolysis in Cerebral Infarction grade 3 recanalization with one pass (Figure 4). The total time from the initial intracranial angiogram to recanalization was 44 minutes. Once complete reperfusion was achieved, the remainder of the diagnostic cerebral angiogram was performed bilaterally. No IIAs were identified, and there was no evidence of dissection or underlying vasculopathy of the right ICA to explain the potential cause of the LVO.

**Figure 5:**
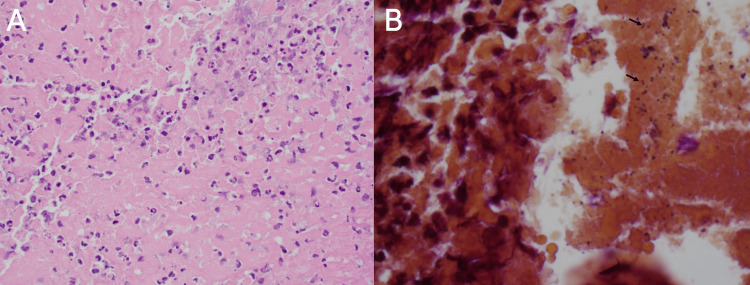
Histopathology of the middle cerebral artery thrombus showing (A) fibrin with abundant neutrophils (hematoxylin and eosin, ×400) and (B) numerous Gram-positive cocci in pairs (arrows, tissue gram stain, ×1000).

The MCA clot removed during thrombectomy was sent for culture to determine if the LVO was caused by an intraprocedural septic embolus from the patient’s underlying endocarditis. The gross appearance of the thrombus was described as a “tan soft tissue fragment” by the pathologist. Histopathological analysis of the MCA thrombus revealed fibrin with abundant neutrophils (Figure 5A). The culture grew group B beta-hemolytic streptococcus, suggesting that the intracranial embolus likely originated from the vegetation on the patient's bioprosthetic aortic valve (Figure 5B).

After the procedure, the patient was able to follow commands on the right side and in the left lower extremity, and she could open her eyes when prompted. She subsequently underwent aortic and mitral valve replacement and remained in the hospital for ongoing cardiovascular and infectious disease management. Institutional review board approval was not required for cases involving fewer than three patients, and verbal patient consent was obtained. The CARE reporting guidelines were followed to ensure comprehensive and standardized reporting practices [8].

## Discussion

Diagnostic techniques, such as cerebral angiograms, are commonly used in the setting of IE due to the substantial risk of IIA and cerebral hemorrhage [9,10]. In this case, a septic embolus was encountered during the diagnostic procedure, which was not visible on the initial craniocervical run, suggesting that the clot was embolized during the procedure. The extracted clot was cultured, confirming it as a septic embolus originating from the patient’s valvular vegetation.

Embolic stroke is a relatively common complication of IE [2,3]. The prognosis for patients with septic stroke secondary to IE is worse compared to those with other stroke etiologies, such as atrial fibrillation or atherosclerotic disease, with mortality rates ranging from 20% to 60% [11,12]. As treatment modalities for endocarditis improve, the prevalence of patients living with endocarditis is also likely to increase [13]. Treatment options for stroke secondary to IE include antibiotic and thrombolytic therapies; however, there are no clear guidelines in the current literature regarding the indications for each treatment strategy [14,15]. Studies suggest that intravenous thrombolysis in IE increases the risk of hemorrhagic complications [15,16], partly due to the presence of mycotic aneurysms, which may rupture after thrombolysis [15]. EVT is now a standard of care for patients with acute ischemic stroke caused by LVO; however, there is limited evidence supporting its safety and efficacy in stroke caused by septic emboli [17]. Systematic reviews and case studies continue to build evidence for EVT in the context of IE [18,19]. A case-control study by Marnat et al. [6] comparing EVT outcomes for stroke due to IE with EVT for stroke secondary to atrial fibrillation found no difference in rates of successful reperfusion, favorable clinical outcome, or symptomatic intracranial hemorrhage. In their cohort, successful reperfusion was achieved in 85.7% of cases, while symptomatic intracranial hemorrhage occurred in 8.0% of cases, suggesting the relative safety and efficacy of mechanical thrombectomy in an infectious setting. A propensity-matched analysis from Germany demonstrated that successful recanalization was achieved in 74.5% of patients with IE and 87.5% of patients with embolic stroke from atrial fibrillation [20]. Similarly, a lower percentage of patients with IE had good functional outcomes at three months (Modified Rankin Scale of 0-2) compared with patients with atrial fibrillation (20.0% vs 43.3%, p = 0.006). However, it is essential to recognize that the clinical prognosis of patients with IE may be worse because of the inherently poor prognosis of IE itself. Additionally, significant advancement in thrombectomy techniques over the past decade may influence outcomes, depending on when the study was conducted and the techniques used. Further research controlling for specific EVT techniques is needed to better understand the safety and effectiveness of EVT for patients with embolic stroke from IE.

## Conclusions

We present a case of LVO from a septic embolus to the right MCA originating from an infected valve, which occurred during diagnostic cerebral angiography. After the rapid intraprocedural identification of the LVO, the occluded vessel was successfully revascularized using EVT. As the literature regarding the efficacy of EVT for LVO secondary to IE continues to evolve, this case adds further evidence in support of EVT.
